# Genome-wide aberrant methylation in primary metastatic UM and their matched metastases

**DOI:** 10.1038/s41598-021-03964-8

**Published:** 2022-01-07

**Authors:** Kyra N. Smit, Ruben Boers, Jolanda Vaarwater, Joachim Boers, Tom Brands, Hanneke Mensink, Robert M. Verdijk, Wilfred F. J. van IJcken, Joost Gribnau, Annelies de Klein, Emine Kilic

**Affiliations:** 1 Department of Ophthalmology, Erasmus MC, Rotterdam, The Netherlands; 2 Department of Clinical Genetics, Erasmus MC, Rotterdam, The Netherlands; 3 Oncode Institute, Department of Developmental Biology, Erasmus MC, Rotterdam, The Netherlands; 4 The Rotterdam Eye Hospital, Rotterdam, The Netherlands; 5 Department of Pathology, Section Ophthalmic Pathology, Erasmus MC, Rotterdam, The Netherlands; 6 Center for Biomics, Department of Cell Biology, Erasmus MC, Rotterdam, The Netherlands

**Keywords:** Metastasis, Eye cancer, DNA methylation

## Abstract

Uveal melanoma (UM) is an aggressive intra-ocular cancer with a strong tendency to metastasize. Metastatic UM is associated with mutations in *BAP1* and *SF3B1,* however only little is known about the epigenetic modifications that arise in metastatic UM. In this study we aim to unravel epigenetic changes contributing to UM metastasis using a new genome-wide methylation analysis technique that covers over 50% of all CpG’s. We identified aberrant methylation contributing to *BAP1* and *SF3B1*-mediated UM metastasis. The methylation data was integrated with expression data and surveyed in matched UM metastases from the liver, skin and bone. UM metastases showed no commonly shared novel epigenetic modifications, implying that epigenetic changes contributing to metastatic spreading and colonization in distant tissues occur early in the development of UM and epigenetic changes that occur after metastasis are mainly patient-specific. Our findings reveal a plethora of epigenetic modifications in metastatic UM and its metastases, which could subsequently result in aberrant repression or activation of many tumor-related genes. This observation points towards additional layers of complexity at the level of gene expression regulation, which may explain the low mutational burden of UM.

## Introduction

Uveal melanoma (UM) is an aggressive malignancy that arises from melanocytes located in the uveal tract of the eye. At the time of diagnosis, only a few patients show metastases, however up to half of the UM will eventually metastasize to other organs. Although the primary tumor can be successfully controlled by surgery or radiation therapy and metastatic risk can be reliably predicted in most patients, there are no effective therapies for metastatic UM^1,2^. Once metastases have been detected disease-related death usually occurs within one year^3^. Metastatic spreading is a complex multi-step process driven by multiple independent (epi)genetic mechanisms. Understanding the specific pathways that initiate and facilitate UM metastasis is essential for the development of a successful treatment.

Metastatic UM is associated with several genetic features, such as loss of chromosome 3 and mutations in BRCA-associated protein 1 (*BAP1*) and splicing factor 3b (*SF3B1*)^4^. UM that harbor a loss of function mutation in *BAP1* often show concurrent loss of chromosome 3, thereby resulting in total loss of the BAP1 protein. *BAP1*-mediated metastasis typically occurs within 5 years after diagnosis, whereas *SF3B1*-mediated metastasis can occur up to 15 years after diagnosis^5,6^. UM that harbor a mutation in eukaryotic transcription initiation factor 1A (*EIF1AX*) rarely metastasize^7^. The genetic alterations that contribute to metastatic spread have been extensively described, however the epigenetic alterations contributing to UM metastasis have been investigated to a lesser extent. Since UM is a disease with relatively few genetic abnormalities^8^, epigenetic regulation might play a pivotal role in *BAP1* and *SF3B1*-mediated metastatic spreading of UM.

In recent years, several studies have focused on identifying aberrant methylation in high metastatic risk, *BAP1*-mutated UM. These studies were performed by using bisulphite conversion of the DNA and subsequently analysed methylation by Sanger sequencing of one specific gene or using an Illumina methylation array chip^8–12^. One shortcoming of the methylation-array is that the design is based on the co-methylation assumption^13^. Probes detect methylation in one CpG and assume that adjacent CpG sites are similarly methylated, which might result in identifying false positives and negatives. Additionally, it has been shown that methylation arrays are not entirely hypothesis-neutral, since the probes are designed to cover CpGs that have been identified as differentially methylated in other studies. Therefore, we analysed the genome-wide methylome of 29 primary UM and 15 UM metastases in an unbiased manner by making use of a recently developed method; MeD-seq^14^. This assay allows sequencing of only methylated DNA by digesting DNA with the DNA-methylation dependent restriction enzyme LpnPI. Subsequently, differentially methylated regions (DMRs) are identified by binning a minimum of ten significantly called (FC > 2 and FDR < 0.5) LpnPI sites in a minimum size of 100 bp. More than 50% of the CpG’s in the human genome are covered, whereas most commonly used techniques such as the Illumina methylation array will detect less than 2% of the CpG’s.

This is the first study to use genome-wide methylation sequencing in metastatic primary UM and its corresponding metastases. By performing an integrated methylomic and transcriptomic approach (Supplementary Fig. 1) we identified potential epigenetic mechanisms contributing to *SF3B1* and *BAP1*-mediated UM metastasis. Additionally, we compared the methylation status in primary UM and its metastases in liver, bone or skin, thereby allowing us to identify metastases-specific DMRs.

## Results

### Global methylation profiles within UM

**Figure 1:**
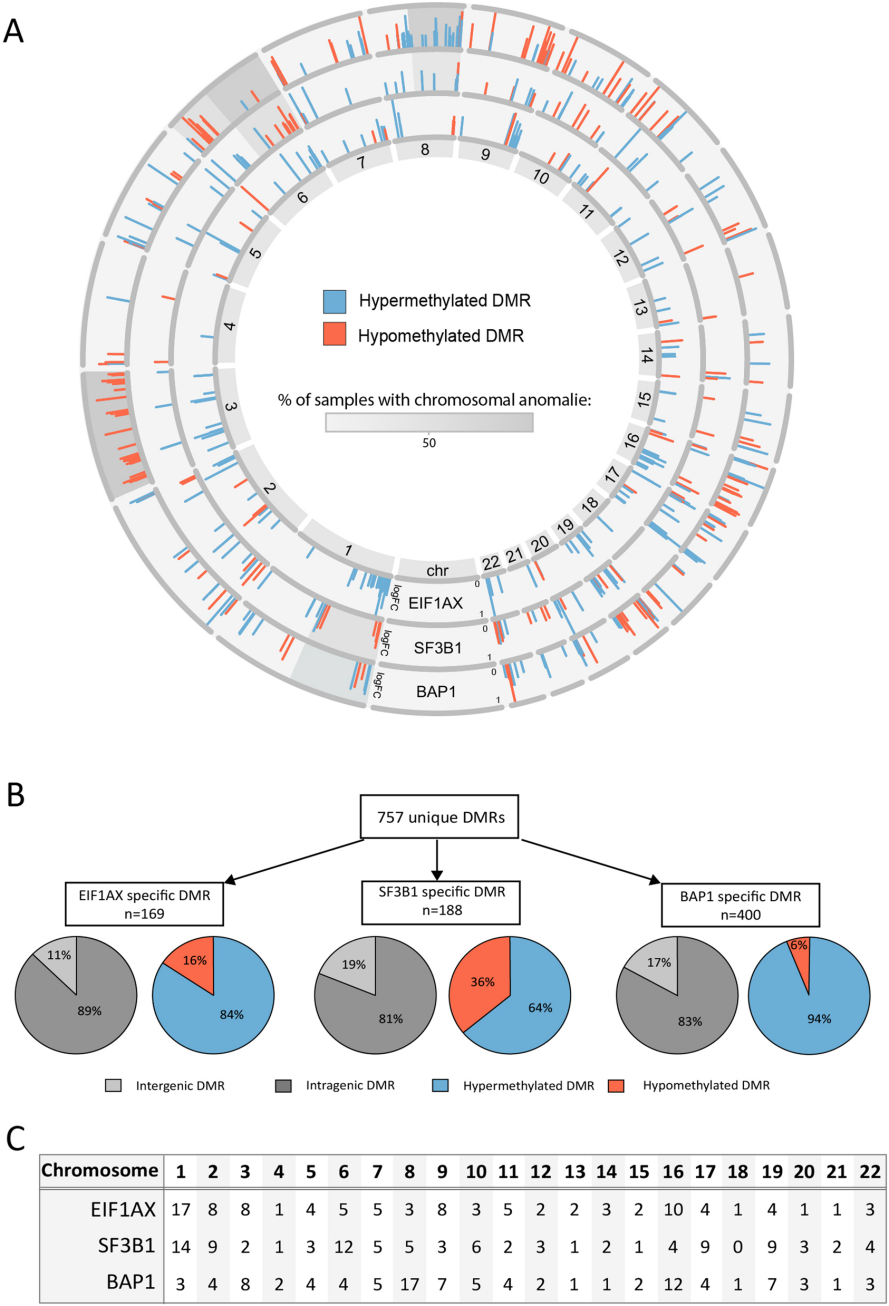
**(A**) All differentially methylated regions (DMR) visualized per chromosome in a donut plot. The height of the bar indicates the log Fold Change (0–1). Chromosomal anomalies are indicated by the grey scale. The outer ring shows the BAP1 unique DMRs, the middle ring the SF3B1 unique DMRs and the inner ring the EIF1AX unique DMRs. (**B)** Quantification of all unique DMRs per group. The genomic location of each DMR is indicated by the grey pie chart, where light grey indicates an intergenic location and dark grey indicates an intragenic location. The amount of methylation of each DMR is shown by the red (hypomethylation) or blue (hypermethylation) pie chart. (**C)** The percentage of DMRs per chromosome is shown for each individual group.

To identify DMRs in metastatic UM, we performed genome sequencing on LpnP1-digested DNA from 29 primary UM samples. Of these 29 samples; 7 were primary UM harboring an *EIF1AX* mutation, 12 *SF3B1*-mutated UM, and 10 UM with a *BAP1* mutation (Table 1). Unique DMRs were identified by comparing the total amount of reads generated per group (i.e. BAP1 vs EIF1AX/SF3B1, SF3B1 vs EIF1AX/BAP1 EIF1AX vs BAP1/SF3B1 ) at every LpnP1-site. Examples of these DMRs are the CTF1 promoter-region and the MNX1 gene body in the *BAP1*-mutated group (Supplementary Fig. 2). 757 unique DMRs (FC > 2) were identified, of which 169 were specific for the *EIF1AX*-mutated UM, 188 for the *SF3B1*-mutated UM and 400 for the *BAP1*-mutated UM (Fig. 1A). The majority of the DMRs identified in these three groups were located in genes, whereas only 11–19% of the DMRs were intergenic (Fig. 1B). Most DMRs showed hypermethylation, as observed in other cancer types, however in the *SF3B1*-mutated group hypomethylation was observed more frequently (36%) than in the other two groups (16% and 6%, respectively). Next, we investigated whether certain chromosomes showed enrichment of DMRs. Chromosome 1, 8 and 16 showed a relatively high number of DMRs, however these chromosomes did not contain the most significant DMRs, i.e. with the highest FC and a FDR < 0,05 (Fig. 1A, 1C).

**Table 1:** Clinical and molecular characteristics of the 29 primary UM samples.

	EIF1AX group	SF3B1 group	BAP1 group
**Number**			
n =	7	12	10
**Metastasis**			
Yes	-	12	10
No	7	-	-
**Disease-free survival (months)**			
Mean + /- SD	145.1 ± 45.1	103.3 ± 50.6	29.5 ± 11
**Chromosome 1**			
1p loss	-	6	4
**Chromosome 3**			
3 loss	-	-	10
**Chromosome 6**			
6q loss	-	7	2
6p loss	1	-	-
6q gain	3	11	1
6p gain	-	-	2
**Chromosome 8**			
8q gain	1	7	9
8p loss	1	1	4

### Functional implications of DNA methylation changes

**Figure 2:**
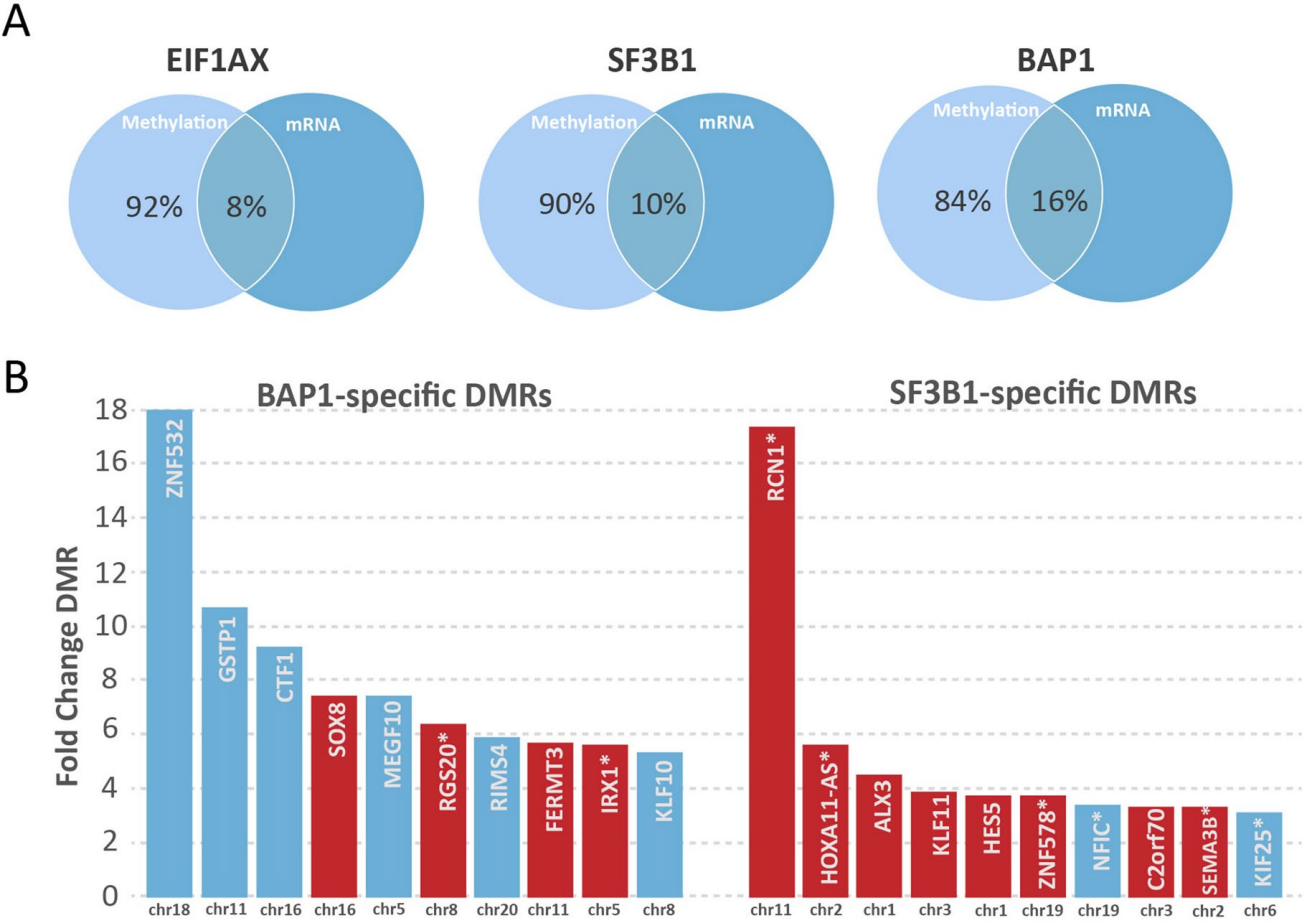
**(A)** The overlap of differential methylation with differential gene expression in the *EIF1AX*, *SF3B1* and *BAP1* group. (**B)** The top ten most significantly methylated regions specific for the *BAP1*-mutated, early metastasizing UM and the SF3B1-mutated, late metastasizing UM. Blue bars indicate downregulated expression, whereas red indicates upregulation of expression. Bars without an asterisk indicate differential methylation in promoters, whereas bars with an asterisk indicate differential methylation in the gene body.

Alterations in the accessibility of DNA by methylation can affect gene expression. In order to determine which genes might be affected by differential methylation in a specific region, each DMR location was matched to the corresponding gene promoter, gene body or CpG island according to the UCSC annotations (Hg38). Since approximately 15% of the DMRs were located outside genes, these could not be matched with expression data. To identify methylation changes associated with significant differential gene expression, we performed an integrative analysis with gene expression data from primary UM samples. DMRs located in the promoter or gene body of a gene were used in this analysis. Hypomethylated promoter and hypermethylated gene body DMRs were integrated with genes that were shown to be significantly upregulated on mRNA level, whereas hypermethylated promoter and hypomethylated gene body DMRs were matched to downregulated genes^15^. Between 8–16% of the DMRs were associated with a change in gene expression (Fig. 2A). All BAP1 and SF3B1-specific DMRs found in primary UM that show association with mRNA expression are listed in Supplementary Table 2 and 3. The most significant DMRs associated with gene expression changes, include several cancer-related genes such as *KLF10*, *GSTP1*, *MEGF10*, *SOX8* and *IRX1*^16–28^ (Fig. 2B).

### Differential methylation in SF3B1 and BAP1-mutated UM metastases

**Figure 3:**
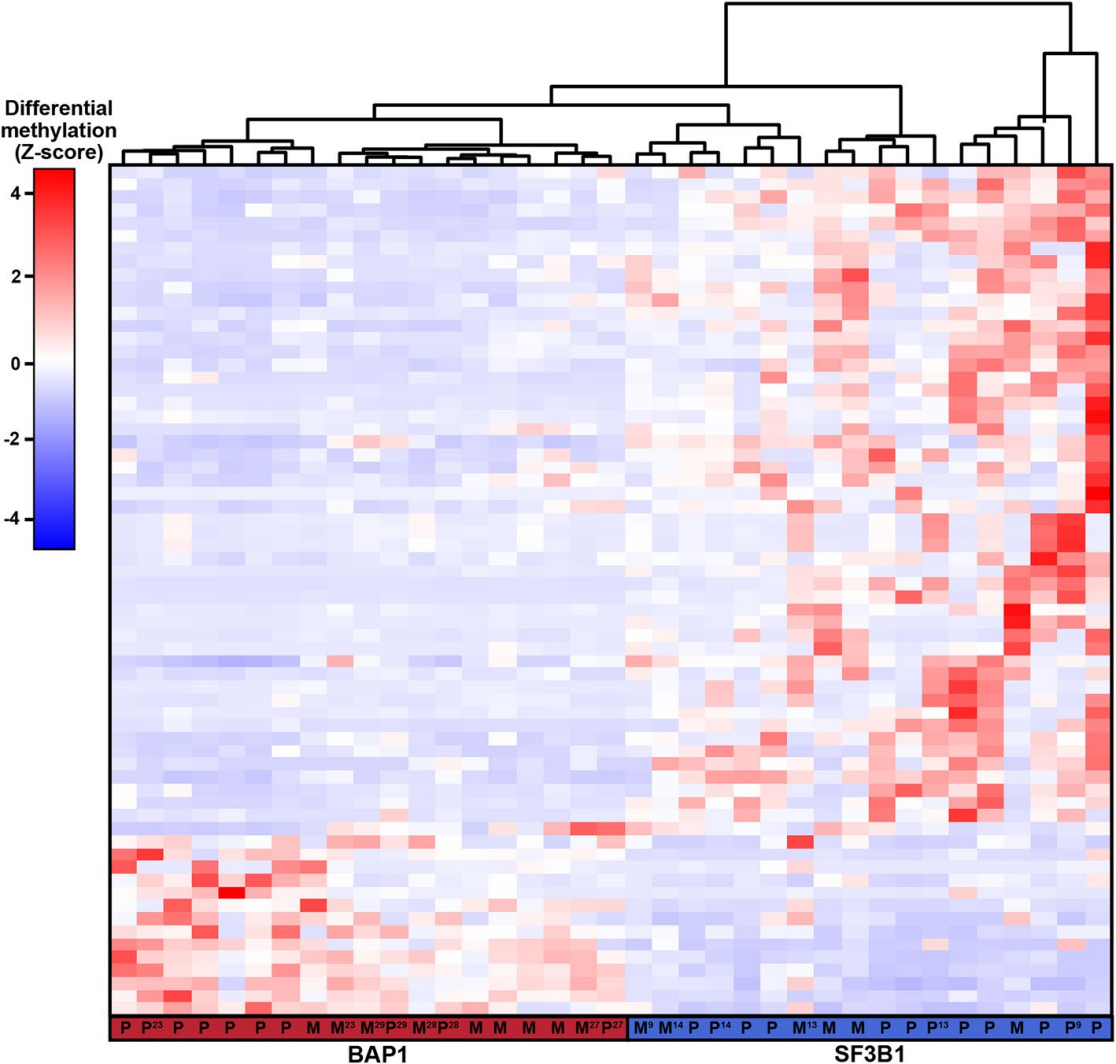
Heatmap visualizing the clustering of primary UM (P) and UM metastases (M) based on DMR with a FC > 5. *SF3B1*-mutated primary UM show a similar methylome as *SF3B1*-mutated metastases and the same is observed for the *BAP1*-mutated primary UM and *BAP1*-mutated metastases. Red indicates hypermethylation, whereas blue indicated hypomethylation of the DMR. Matched primary UM and metastases samples are marked by their sample number (UM23, UM27, UM28, UM29, UM9, UM13, UM14).

The methylome of 15 UM metastases was investigated to survey if the identified DMRs were present in UM metastases from liver, skin or bone as well. Tumor cell percentages were determined on HE stained tissue sections by an experienced ocular pathologist and ranged from 30–98% (Supplementary Table 1). Despite this varying tumor cell content and different tissues, hierarchical clustering using DMRs with a FC > 5 showed that UM metastases clustered together with either *BAP1* or *SF3B1*-mutated UM implicating that they showed the same secondary driver signature (Fig. 3).

**Figure 4:**
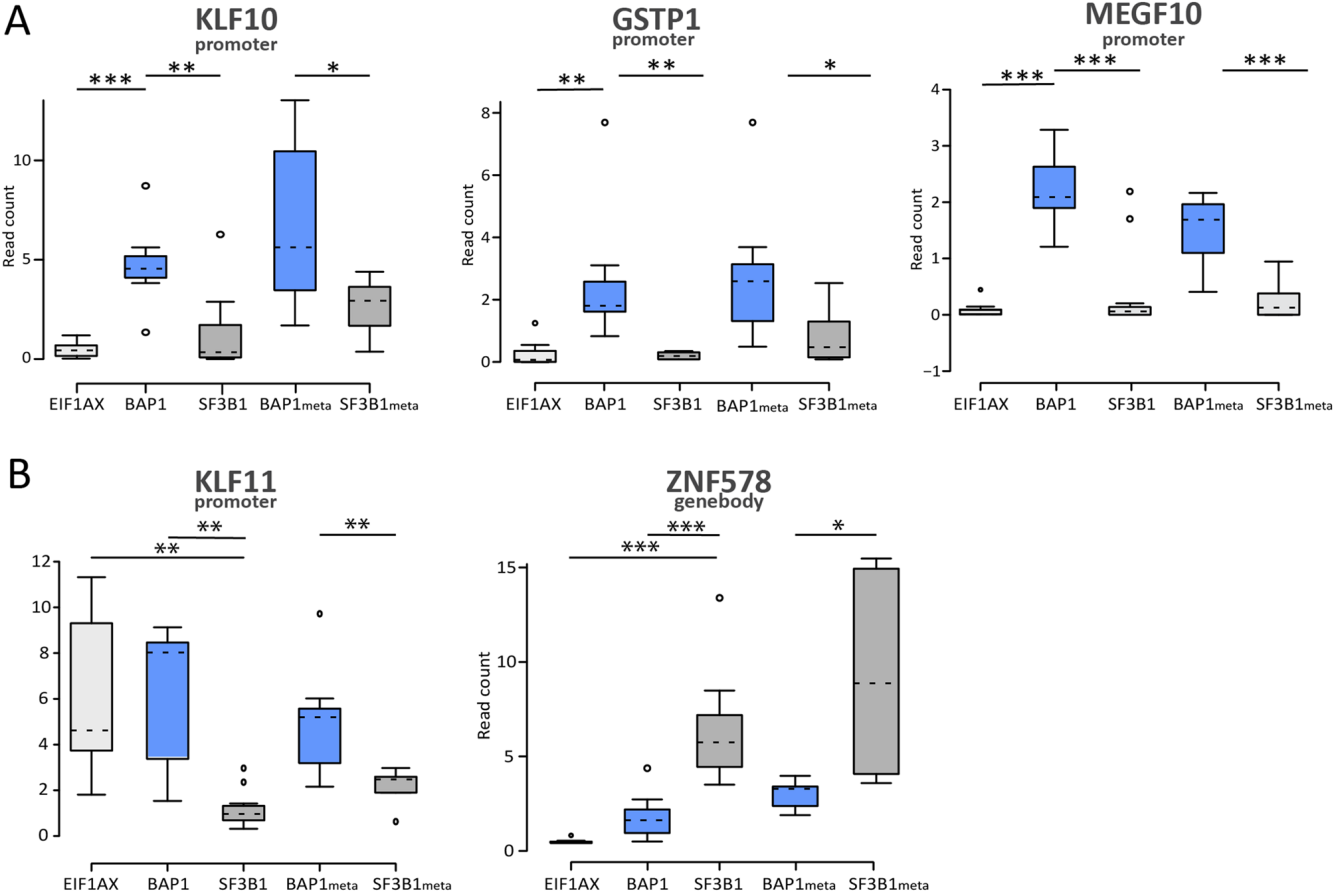
Boxplots showing the normalized read count level, which indicates the level of methylation, for (**A)** Differentially methylated genes in BAP1-mediated metastasis. (**B)** Differentially methylated genes in SF3B1-mediated metastasis. Significance differences between the groups are indicated on the top. *P < 0.05, **P < 0.01, ***P < 0.001.

**Figure 5:**
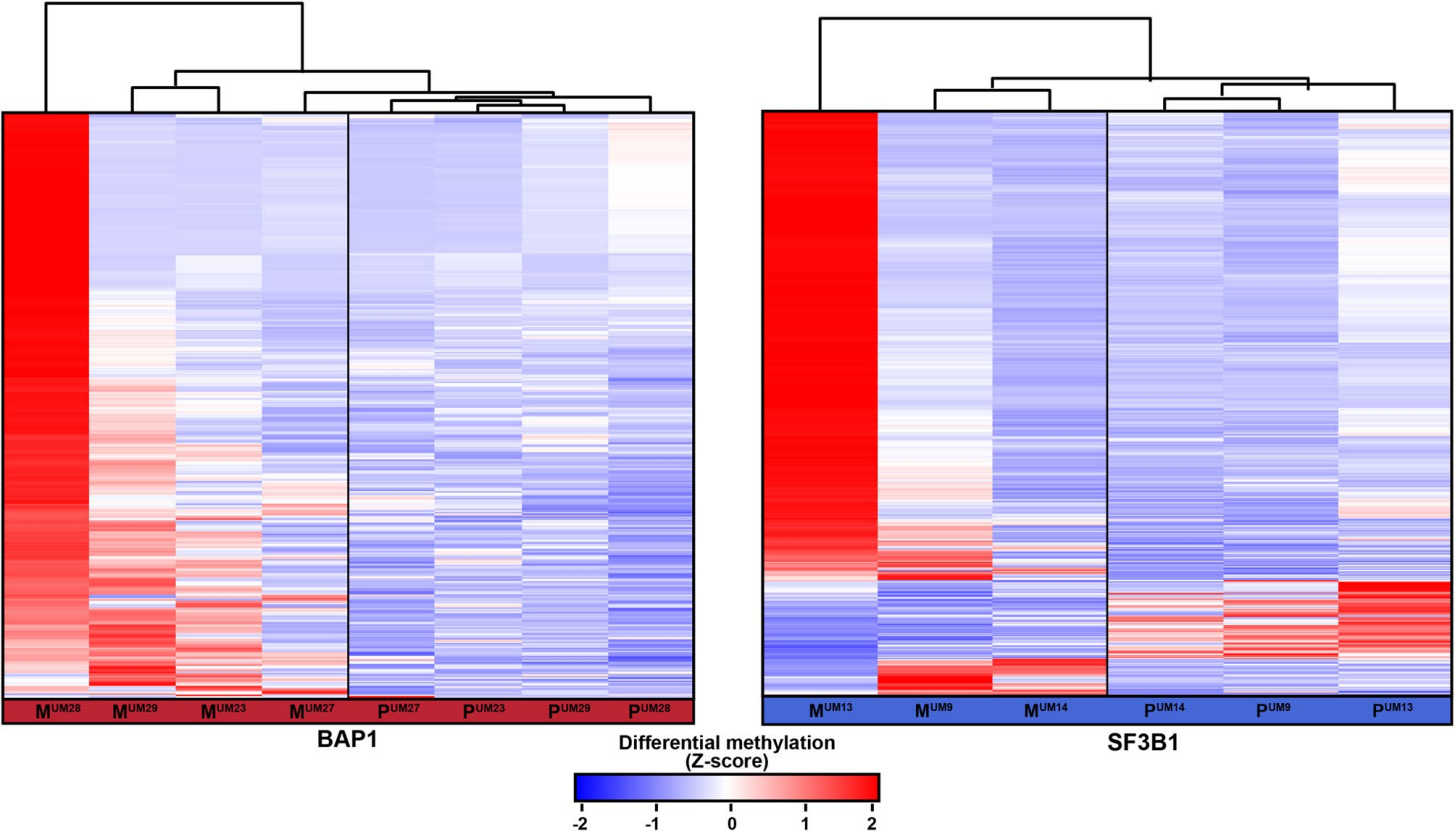
Heatmap visualizing the clustering of the 3 *SF3B1*-mutated matched primary UM and UM metastases and 4 *BAP1*-mutated matched primary UM and UM metastases based on DMR with a FC > 2. Red indicates hypermethylation, whereas blue indicated hypomethylation. Matched primary UM and metastases samples are marked by their sample number (UM23, UM27, UM28, UM29, UM9, UM13, UM14).

Seven matched primary tumor and metastases sets were included and clustered in proximity of each other. The DMRs that showed correlation with gene expression were also surveyed in the UM metastases. Not all DMRs showed differential methylation in the metastases samples as well. However, we did observe differential methylation of the tumor suppressor genes *KLF10*, *GSTP1* and *MEGF10* and several other genes in all metastases samples (Fig. 4 and supplementary Fig. 3). In paired primary/metastases analysis, a significant number of new DMRs (> 1000) could be identified in distant metastases, which were absent in their corresponding primary tumors. Hierarchical clustering showed that metastases samples clustered together (Fig. 5), interestingly we did not find any significant DMRs (FC > 2) that were shared among the different *SF3B1* or *BAP1*-mutated primary/metastases sets.

## Discussion

Developing a successful treatment for metastatic UM remains one of the most significant challenges in UM research. While current UM research has primarily focused on the genetic factors contributing to UM metastasis, here we aim to elucidate the epigenetic landscape of metastatic UM. We performed MeD-seq, a novel genome-wide sequencing technique^14^ with higher coverage and improved distribution compared to most other techniques, to explore associations between aberrant methylation and *BAP1* and *SF3B1*-mutated mediated metastasis. We identified 757 genes affected by differential methylation, of which the majority showed hypermethylation. Interestingly, another study compared healthy tissue to tumor tissue and found a similar level of hyper and hypomethylation^29^, suggesting that hypomethylation is mainly important at the start of tumorigenesis . Of these 757 differentially methylated genes, 188 were found in *SF3B1*-mutated UM and 400 in the *BAP1*-mutated UM. This shows DNA methylation is most disrupted in *BAP1*-mutated UM, as also observed by others^9^, however *SF3B1*-mutated UM also showed significant levels of aberrant methylation and both might therefore profit from treatments regulating methylation activity. We do not exclude the possibility of a fourth group since tumors with non-recurrent mutations might exhibit different methylation patterns than UM with a mutation in *EIF1AX*, *SF3B1* or *BAP1*.

Integrating methylation data with expression data showed that 8–16% of the DMRs is associated with aberrant gene expression. Approximately 15% of the identified DMRs are located outside genes and could therefore not be matched with expression data. Since our goal was to identify DMRs with functional implications, we only included genes that showed significant differential expression (FC > 1.5 and FDR < 0.05). Our obtained results correspond to what is found in other studies; where it was shown that DNA methylation is not limited to active genes, but is often targeting silent genes. DNA methylation ensures that decisions made by transcription factors are stabilized and transcription is precise and robust but limited to the sub-set of genes required^30^.

UM is known to harbor several chromosomal alterations, such as loss of chromosome 3 and gain of chromosome 6p and 8q. Chromosome alterations can affect the MeD-seq read counts and these counts could therefore be used to detect chromosomal abnormalities. However, they can also result in the detection of false-positive DMRs. Because the methylation data is integrated with gene expression data we omitted the DMRs that do not have a significant impact on gene expression and therefore these false-positive DMRs were also removed from our analysis. Loss of BAP1 expression is an important event in the development of high risk UM and it was suggested by Field et al. that this biallelic loss of BAP1 results into extensive methylomic remodelling, including a dense cluster of hypermethylated genes on chromosome 3^9^. A similar observation was witnessed by Ness et al. where methylation data obtained from 8 UM samples showed clustering based on chromosome 3 status rather than clustering based on risk of metastasis^31^. We also detected a number of DMRs on chromosome 3, however the most significant DMRs that resulted in expression changes were found on other chromosomes.

In *BAP1*-mutated tumors, we observed increased promoter methylation in the tumor suppressor genes *MEGF10, GSTP1* and *KLF10.* These genes showed significant differential methylation in the metastases samples as well. Interestingly, we also observed an association between methylation levels of these genes and disease-free survival. Although these genes are not directly involved in the BAP1-related pathways, an indirect effects of BAP1 on *MEGF10, GSTP1* and *KLF10* might explain this association. Multiple EGF like domains 10 (*MEGF10*) encodes for a transmembrane protein that is highly expressed in the neural tube during early development. It regulates cell migration and adhesion, such as during patterning of retinal neurons^32^. Recently it has been shown that *MEGF10* functions as an important tumor suppressor gene and is often epigenetically repressed in other cancer types, including high-risk neuroblastoma^33,34^. Glutathione S-transferase Pi 1 (*GSTP1*) is a gene that regulates lipid and glycolytic metabolism in a cell^16^. It can regulate oncogenic signalling pathways by activating glyceraldehyde-3-phosphate dehydrogenase and it has been reported that the *GSTP1* promoter is often hypermethylated in tumors^17,18^.

Another tumor suppressor gene that shows aberrant methylation is Kruppel-like factor 10 (*KLF10*). KLF10 is a DNA-transcription regulator that binds GC-rich sequences in gene promoters to inhibit growth and initiate apoptosis through TGFβ-signaling. The TGFβ-signaling pathway is known to play an important role in the maintenance of tissue homeostasis by regulating proliferation and apoptosis. Several studies have described a putative tumor suppressor role for KLF10^19–21^. Epigenetic repression of *KLF10* has been observed to correlate with poor prognosis in pancreatic cancer^22^. Interestingly, *SF3B1*-mutated UM showed upregulation of several transcription factors, such as KLF11. As mentioned previously, upregulation of certain genes through differential methylation might be a direct or indirect consequence of aberrant splicing. Additionally, it has been shown that despite the fact that KLF10 and KLF11 are family members showing a high similarity in their DNA-domains, they have different effects on transcription regulation. KLF10 and KLF11 were originally introduced as transcriptional repressors, however several studies have shown that they can also function as transcriptional activators depending on the cellular context^23^. It has been described for example that KLF11 promotes invasion and migration in gastric cancer through activation of Twist1^24^.

The most significant DMR in *BAP1*-mutated primary UM was observed for the zinc-finger transcription factor ZNF532 (Supplementary Table 2); unfortunately, no specific description is available in literature about the function of this gene. Given the strong significance, this might be an interesting gene for future UM research. Other genes that caught our interest are *SOX8*, *RGS20* and *IRX1*. These genes are known to be involved in carcinogenesis and we observed demethylation of these genes in the *BAP1*-mutated primary UM. SRY-box 8 (SOX8) and Iroquois homeobox protein 1 (IRX1) are both transcription factors that are involved during embryonal development. Field et al. also observed a similar stem-cell like phenotype when investigating the methylome of *BAP1*-mutated UM^9^. Overexpression of these genes can contribute to cancer by activating genes that induce a more stem cell-like properties in UM cells, which eventually can result in metastasis. Interestingly, metastasizing hepatocellular carcinomas and squamous cell carcinomas show higher expression of *SOX8* too^25,26^. *IRX1* is upregulated in metastasized osteosarcoma^28^ and in leukaemia, where it predicts worse outcome^27^.

Several differentially methylated regions in UM were identified in previous studies, such as *p16*^*in4a*^^35^ *TIMP3*^36^*, RASSF1A*^10,12^*, TERT*^11^*, LZTS1*^37^*, EFS*^38^*, PRAME*^39^ and *RAB31*^29^*.* Of these previously identified genes *p16ink*, *TERT*, *LZTS1*, *EFS* and *PRAME* were found to be differentially methylated in our analysis as well (data available upon request). However, none showed differential gene expression between the EIF1AX, SF3B1 or BAP1 subgroups. A recent whole genome methylation study from Robertson and colleagues^8^, identified several genes as differentially methylated, such as *PVT1*, *ENPP2*, *C2orf70*, *DGKB* and *PACSIN3*. In our analysis we only identified ENPP2 and C2orf70 to be differentially methylated. These differences can be explained by the techniques used to detect DMRs. In our study, DMR calling is based on a large number of CpGs per gene, whereas HM450 arrays identify differential methylation based on few CpGs per gene. DNA methylation rarely takes place in isolated CpGs and is more likely to affect continuous gene regions containing many CpGs as interrogated by MeD-seq.

Not every DMR that resulted in differential expression could be confirmed in the UM metastases samples. However, this does not mean that they do not contribute to UM metastasis. Firstly, our metastases samples did not consist of a pure population of tumor cells. The non-tumor cells present in UM metastases samples could interfere in the methylation analysis, therefore causing only detection of the highly significant DMRs. Secondly, metastasized UM cells lose a part of their phenotype once they are fully integrated in the hosting organ^40^. Thirdly, as previously described by Shain et al.^41^ UM metastases might arise from one or a few cell clones that disseminated early in the development of UM and therefore evolved differently than the primary tumor. We observed this in both *SF3B1* as *BAP1*-mutated metastases.

When comparing primary tumors with their corresponding metastases we observed a large number DMRs, however none of these DMRs were shared among the *SF3B1* and *BAP1*-mutated sets . Hierarchical clustering did overall show more similarity between metastases, compared to the primary UM. This implies that most of the methylation events occurring in the metastases are likely patient specific and subject to several processes such as time of metastatic dissemination and progression. Some of the differential methylation found in metastases might also be occurring randomly, rather than driving the colonization of metastatic UM cell. Unfortunately, none of these methylation events could be directly linked to early or late SF3B1-mediated metastasis. Although this could also be explained by the small number of samples. In order to elucidate the biological relevance of specific methylation events in UM metastases, it is crucial to include more matched primary and metastatic tumors.

The identified differentially methylated regions might be an interesting target for liquid biopsies in UM. Detecting methylated cell-free DNA in the circulation of UM patients could be an indicator for high risk UM, but another important advantage is that it might provide us with an interesting therapeutic target^42,43^. Unlike mutations in the DNA, methylation on the DNA can be easily reversed by (de)methylating agents. By removing excessive methylation, tumor suppressor genes can be reactivated in tumors which reduces proliferation and migration of tumor cells. Our findings can be interpreted in two ways. It is possible that the mutations in *EIF1AX*, *SF3B1* and *BAP1* initiate de novo methylation and demethylation and thereby promote a series of gene expression changes. Alternatively, the aberrant methylation may arise early in the oncogenic transformation of uveal melanocytes, in which case targeting the methylation might strike the Achilles heel of UM.

## Conclusion

We show aberrant DNA methylation in several novel genes, such as *KLF10*, *GSTP1* and *MEGF10*, that correlate with altered gene expression in primary UM and its metastases. Matched primary and metastatic tumors show a distinct methylation pattern, but no commonly shared DMRs were identified. Therefore, we propose potential early onset epigenetic mechanisms in BAP1 and SF3B1-mediated metastasis identified by using a novel approach integrating MeD-seq derived methylation data and gene expression data. Confirming our results in a larger cohort and subsequent biological analysis of the proteins encoded by these aberrantly methylated genes may lead to a better understanding of UM metastasis.

## Methods

### Sample collection

Twenty-nine primary UM samples and 15 UM metastases samples, including seven pair-matched samples, were selected from our Rotterdam Ocular Melanoma Study Group (ROMS) database. Primary tumors were obtained from patients that underwent enucleation as primary therapy at the Department of Ophthalmology, Erasmus MC and the Rotterdam Eye Hospital. Fifteen metastases were obtained from 10 UM patients and were resected from liver, skin, pancreas or bone. All primary tumors were collected as fresh specimens, whereas the UM metastases samples included two formalin-fixed, paraffin-embedded (FFPE) and 13 fresh specimens (Supplementary Table 1). BAP1 immunohistochemistry, copy number profiling and mutation detection was performed as described previously^44,45^. This study was approved by the Medical Ethics Review Committee of the Erasmus MC/Erasmus University Rotterdam (MEC-2009–375), all patients signed an informed consent and the study was performed according to the guidelines of the Declaration of Helsinki.

### DNA isolation and processing

After enucleation, a part of the primary tumor was extracted and DNA was isolated from fresh or fresh-frozen tumor by using the QIAmp DNA mini kit (Qiagen, Hilden, Germany) according to the manufacturer’s protocol. All FFPE samples were de-paraffinized and hematoxylin-stained prior to DNA isolation. FFPE-sections were micro-dissected by manually scraping the metastatic UM cells from the sections and sodiumthiocyanate was used to remove the crosslinks formed after fixation. Subsequently, DNA was isolated by using the QIAmp DNA mini kit (Qiagen). DNA concentrations were measured using the Quant-iT Picogreen assay kit (Thermo Fisher Scientific, Grand Island, NY, USA) as described by the manufacturer. The total DNA-yield from all samples included in this study ranged from the minimum 50 ng to 8 ug.

### MeD-seq sample preparation

LpnPI (New England Biolabs, Ipswhich, MA, USA) digestions were carried out on DNA samples according to the manufacturer’s protocol. Reactions contained 50 ng in 10 μl volume and digestion took place overnight in the absence of enzyme activators. Digests of genomic DNA with LpnPI resulted in snippets of 32 bp around the fully-methylated recognition site that contains CpG. These short fragments were either purified on TBE gel before preparation or purified by Pippin system gel after preparation. Gel purification was performed with 10% TBE gels using the Xcell SureLock system. Sixty microliters of each sample was loaded on the gel, leaving at least one empty well between samples. After running, gels were stained by ethidiumbromide and scanned on a Typhoon Trio. DNA was cut out based on ladder sizes at 30–40 bp and extracted from gel using gelbreaker tubes and centrifugation. DNA was washed with 70% EtOH and dissolved in 10 mM Tris–HCl (pH 8.5). The DNA concentration was determined by the Quant-iT High-Sensitivity assay (Life technologies, Carlsberg, CA, USA; Q33120) and 50 ng ds DNA was prepared using the ThruPlex DNA-seq 96D kit (Takara Bio Inc, Kusatasu, Japan). For Pippin gel purification, twenty microliters of amplified end product was purified on a Pippin HT system with 3% agarose gel cassettes (Sage Science, Beverly, MA, USA). Stem-loop adapters were blunt end ligated to repaired input DNA and amplified (4 + 10 cycles) to include dual indexed barcodes using a high fidelity polymerase to yield an indexed Illumina NGS library (Illumina, San Diego, CA, USA). Multiplexed samples were sequenced on Illumina HiSeq2500 systems for single read of 50 base pairs according to the manufacturer’s instructions. Dual indexed samples were demultiplexed using Bcl2fastq Software Version 2.20.0.422 available at https://emea.support.illumina.com/downloads/bcl2fastq-conversion-software-v2-20.html (Illumina).

### MeD-seq data processing

Data processing was carried out as described before^14^ using specifically created scripts in Python Version 2.7.5 (Python Software Foundation available at http://www.python.org). In short, raw FASTQ files were subjected to Illumina adaptor trimming and reads were filtered based on LpnPI restriction site occurrence between 13–17 bp from either 5’ of 3’ end of the read. Reads that passed the filter were mapped to hg38 using bowtie 2.1.0. Multiple and unique mapped reads were used to assign read count scores to each individual LpnPI site in the hg38 genome. BAM files were generated using SAMtools for visualization. Gene and CpG island annotations were downloaded from UCSC (hg38). Genome wide individual LpnPI site scores were used to generate read count scores for the following annotated regions: transcription start site (TSS) (1 kb before and 1 kb after), CpG island and gene body (1 kb after TSS until TES).

### MeD-Seq data analysis

Data analysis was carried out in Python Version 2.7.5 using custom Python Scripts (Packages Matplotlib en Scipy). DMR detection was performed between two datasets (i.e. BAP1 vs EIF1AX/SF3B1, SF3B1 vs EIF1AX/BAP1 EIF1AX vs BAP1/SF3B1) containing the regions of interest (TSS, gene body or CpG islands) using the Chi-Squared test on read counts. Significance was called by either Bonferroni or FDR using the Benjamini–Hochberg procedure. Differentially methylated regions were used for hierarchical clustering (method = ”complete”, metric = ”cityblock”), the data was normalizing using RPM (reads per million) and a Z-score was calculated and shown in the heatmaps. In addition, a genome wide sliding window was used to detect sequentially differentially methylated LpnPI sites (by selecting a single LpnPI site and adding a maximum of 50 neighboring LpnPI sites either up- or downstream within a 1000 distance from the initial LpnPI site. Statistical significance was called between LpnPI sites in predetermined groups using the Chi-squared test and Bonferroni correction. Neighbouring significantly called LpnPI sites were reported, DMR threshold was set at a minimum of ten LpnPI sites, a minimum size of 100 bp and either twofold change in read counts for the general analysis or fivefold change when we performed hierarchical clustering. Overlap of genome wide detected DMRs was reported for TSS, CpGisland or gene body regions. Annotation overlap for DMRs detected were called on any overlap (partial or total) based on previous described TSS and gene body region boundaries and CpG Island annotations.

### mRNA sequencing

Total RNA was extracted from snap-frozen tumor samples using the Qiagen miRNeasy isolation kit (Qiagen) according to the manufacturer’s instructions. mRNA sequencing was performed as previously described^46^. Genes were considered to be differentially expressed if they had at least a log2FC of 1.5 and FDR < 0.05. All mRNA analyses were performed using R statistical environment Version 3.3.3, available at http://www.R-project.org (R-Core team, Vienna, Austria).

### Ethics approval and consent to participate

This study was performed according to the guidelines of the Declaration of Helsinki and approved by the local Medical Ethics Committee (MEC-2009–375, 12 November 2009). Informed consents were obtained at the time of diagnosis.

## Supplementary Information

Supplementary Information.

## Data Availability

Data available upon request.
