# Supplementary material for: Genome-wide aberrant methylation in primary metastatic UM and their matched metastases
Source: Sci Rep. 2022 Jan 7;12:42. doi: 10.1038/s41598-021-03964-8 (PMC8742000; doi:10.1038/s41598-021-03964-8)
Supplement: Supplementary file 1 — Supplementary Information. [file 41598_2021_3964_MOESM1_ESM.docx]

**Aberrant methylation in primary metastatic UM and their matched metastases**Kyra N Smit^1,2^, Ruben Boers^3^, Jolanda Vaarwater^1,2^, Joachim Boers^3^, Tom Brands^2^, Hanneke Mensink^4^, Robert M Verdijk^5^, Wilfred FJ van IJcken^6^, Joost Gribnau^3^, Annelies de Klein^2^ and Emine Kilic^1*^*^1^ Department of Ophthalmology, Erasmus MC, Rotterdam, The Netherlands; ^2^ Department of Clinical Genetics, Erasmus MC, Rotterdam, The Netherlands; ^3^ Oncode Institute, Department of Developmental Biology, Erasmus MC, Rotterdam, The Netherlands; ^4^ The Rotterdam Eye Hospital, Rotterdam, The Netherlands; ^5^Department of Pathology, Section Ophthalmic Pathology, Erasmus MC, Rotterdam, The Netherlands; ^6^ Center for Biomics, Department of Cell Biology, Erasmus MC, Rotterdam, The Netherlands*

**
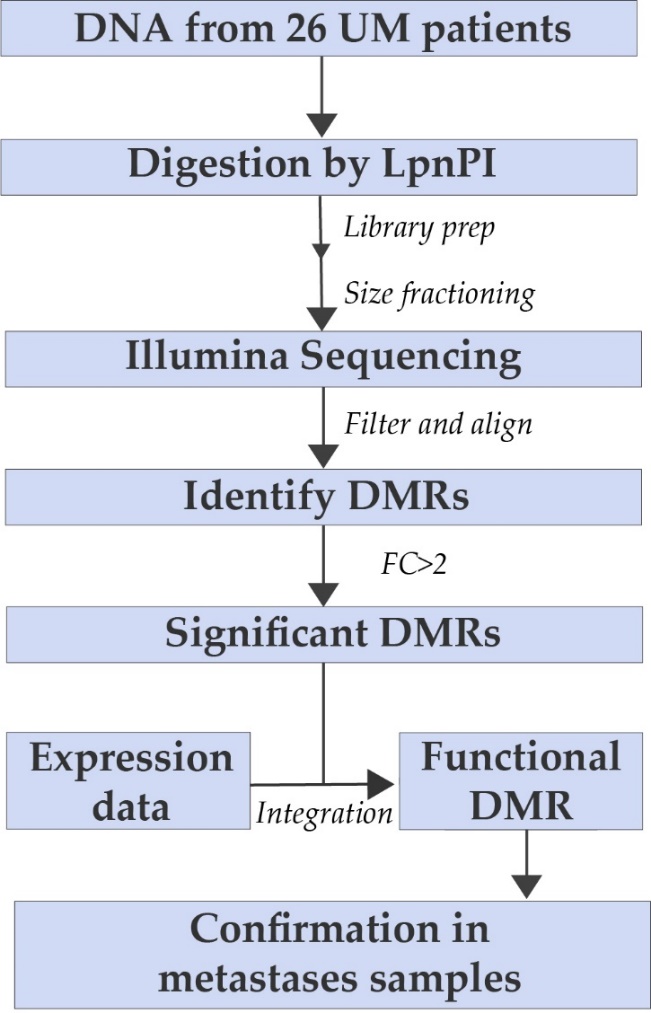
Supplementary Figure 1.** Flowchart showing the different steps taken to identify methylation associated with SF3B1 and BAP1-mediated metastases

|  | **Primary or meta** | **Mutation** | **DFS  (months)** | **Metastasized** | **BAP1 IHC** | **Chr 1** | **Chr 3** | **Chr 6** | **Chr 8** | **Tumor cell percentage** | **Location** | **Material** |
| --- | --- | --- | --- | --- | --- | --- | --- | --- | --- | --- | --- | --- |
| **UM1** | Primary | *EIF1AX* | 188,4 | N | Positive |  |  | +p |  | ~ 100% | Eye | Fresh |
| **UM2** | Primary | *EIF1AX* | 230,7 | N | Positive |  |  |  | + | ~ 100% | Eye | Fresh |
| **UM3** | Primary | *EIF1AX* | 218,1 | N | Positive |  |  |  |  | ~ 100% | Eye | Fresh |
| **UM4** | Primary | *EIF1AX* | 231,7 | N | Positive |  |  |  |  | ~ 100% | Eye | Fresh |
| **UM5** | Primary | *EIF1AX* | 111,9 | N | Positive |  |  | + |  | ~ 100% | Eye | Fresh |
| **UM6** | Primary | *EIF1AX* | 141,7 | N | Positive |  |  | +p |  | ~ 100% | Eye | Fresh |
| **UM7** | Primary | *EIF1AX* | 73,2 | N | Positive |  |  | + |  | ~ 100% | Eye | Fresh |
| **UM8** | Primary | *SF3B1* | 23.2 | Y | Positive | +q |  | +p/-q | -p | ~ 100% | Eye | Fresh |
| **UM9** | Primary | *SF3B1* | 33.4 | Y | Positive |  |  | +p/-q | +q | ~ 100% | Eye | Fresh |
|  | Meta | *SF3B1* |  |  | Negative | ? | ? | ? | ? | 72% | Bone | FFPE |
| **UM10** | Primary | *SF3B1* | 51,9 | Y | Positive | -p | - | +p |  | ~ 100% | Eye | Fresh |
| **UM11** | Primary | *SF3B1* | 102,6 | Y | Positive | -p |  | +p | +q | ~ 100% | Eye | Fresh |
| **UM12** | Primary | *SF3B1* | 129,6 | Y | Positive |  |  |  | +q | ~ 100% | Eye | Fresh |
| **UM13** | Primary | *SF3B1* | 120,3 | Y | Positive | -p |  | +p/-q | + | ~ 100% | Eye | Fresh |
|  | Meta | *SF3B1* |  | Y | Positive | -p |  |  |  | 30% | Liver | Fresh |
| **UM14** | Primary Meta | *SF3B1*  *SF3B1* | 144,7 | Y | Positive | -p  -p |  | +p/-q +p/-q |  | ~ 100% 94% | Eye Liver | Fresh FFPE |
| **UM15** | Primary | *SF3B1* | 83,2 | Y | Positive | -p |  | +p/-q |  | ~ 100% | Eye | Fresh |
| **UM16** | Primary | *SF3B1* | 105,1 | Y | Positive | +q | -q | +p | +q | ~ 100% | Eye | Fresh |
| **UM17** | Primary | *SF3B1* | 132,2 | Y | Positive |  | -q | +p/-q |  | ~ 100% | Eye | Fresh |
| **UM18** | Primary | *SF3B1* | 193,6 | Y | Positive |  |  | +p | +q | ~ 100% | Eye | Fresh |
| **UM19** | Primary | *SF3B1* | 131,2 | Y | Positive | -p |  | +p/-q | + | ~ 100% | Eye | Fresh |
| **UM20** | Primary | *BAP1* | 54,4 | Y | Negative |  | - |  |  | ~ 100% | Eye | Fresh |
| **UM21** | Primary | *BAP1* | 32,4 | Y | Negative | -p | - |  | +q | ~ 100% | Eye | Fresh |
| **UM22** | Primary | *BAP1* | 15,9 | Y | Negative |  | - |  | -p/+q | ~ 100% | Eye | Fresh |
| **UM23** | Primary | *BAP1* | 26,9 | Y | Negative |  | - |  | -p/+q | ~ 100% | Eye | Fresh |
|  | Meta | NE |  |  | Negative |  | - |  | -p/+q | 70% | Liver | Fresh |
| **UM24** | Primary | *BAP1* | 26,1 | Y | Negative |  | - |  | -p/+q | ~ 100% | Eye | Fresh |
| **UM25** | Primary | *BAP1* | 21,4 | Y | Negative | -p | - |  | +q | ~ 100% | Eye | Fresh |
| **UM26** | Primary | *BAP1* | 27,1 | Y | Negative | -p | - |  | +q | ~ 100% | Eye | Fresh |
| **UM27** | Primary  Meta | *BAP1*  *BAP1* | 22,9 | Y Y | Negative Negative |  | - - | +p/-q +p/-q | +q -p/+q | ~ 100% 70% | Eye Liver | Fresh Fresh |
| **UM28** | Primary Meta | *BAP1 BAP1* | 23 | Y | Negative Negative |  | - - |  | -p/+q -p/+q | ~ 100% 50% | Eye Liver | Fresh Fresh |
| **UM29** | Primary Meta | *BAP1 BAP1* | 53,9 | Y | Negative Negative |  | - - |  |  | ~ 100% 52% | Eye Liver | Fresh Fresh |
| **UM30** | Meta | SF3B1 | 98 | Y | Positive | -p/+q | +p/-q | -q | -p | 96% | Liver | Fresh |
|  | Meta | SF3B1 |  |  | Positive | -p/+q | +p/-q | -q | -p | NE | Liver | Fresh |
|  | Meta | SF3B1 |  |  | Positive | -p/+q | -q | -q | -p | NE | Pancreas | Fresh |
| **UM31** | Meta | BAP1 | 16,3 | Y | Negative | -p | - |  | +q | 70% | Liver | Fresh |
|  | Meta | BAP1 |  |  | Negative |  | - |  | +q | 70% | Skin | Fresh |
| **UM32** | Meta | BAP1 | 3,2 | Y | Negative | -p | - | -q | -p/+q | 76% | Liver | Fresh |
|  | Meta | BAP1 |  |  | NE | -p | - | -q | -p/+q | 62% | Skin | Fresh |
|  | Meta | BAP1 |  |  | NE | -p | - | -q | -p/+q | 80% | Skin | Fresh |

**Supplementary Table 1.** Clinical and molecular characteristics of the 29 primary UM and 15 UM metastases samples
NE; not evaluated

**Supplementary Figure 2.** *CTF1* promoter hypermethylation and *MNX1* genebody hypomethylation in *BAP1*-mutated UM compared to *SF3B1* and *EIF1AX*-mutated UM. From top to bottom the following samples are shown: UM1 – UM5, UM8 – UM12 and UM21 – UM25.

**
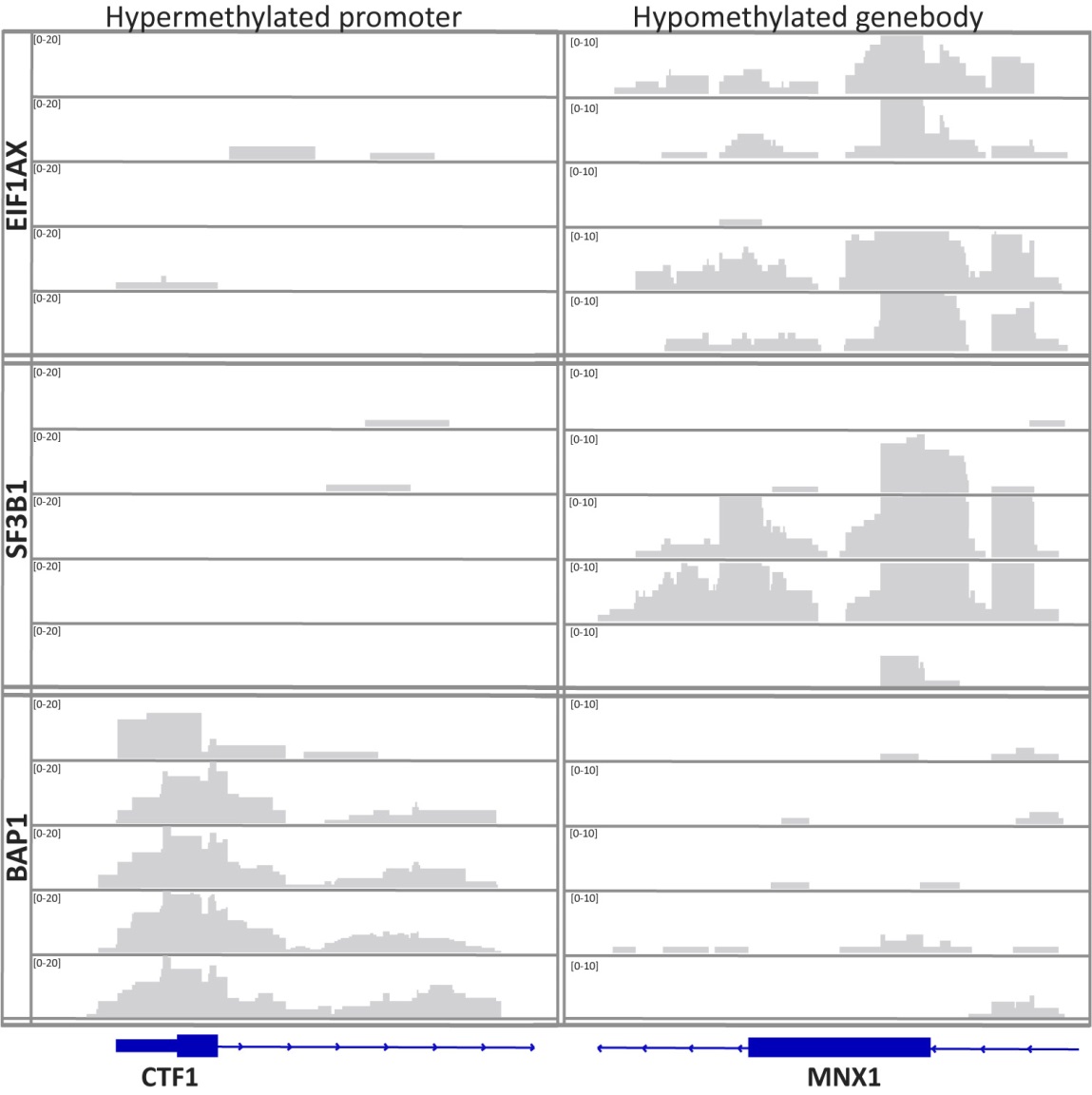
**

**Supplementary Table 2.** BAP1-specific DMRs that cause a significant gene expression change

| **Gene** | **Chromosome** | **Location DMR** | **Type of methylation** | **FC DMR** | **Expression** |
| --- | --- | --- | --- | --- | --- |
| **ZNF532** | 18 | Promoter | Hypermethylation | 17.6 | Downregulated |
| **GSTP1** | 11 | Promoter | Hypermethylation | 10.3 | Downregulated |
| **CTF1** | 16 | Promoter | Hypermethylation | 8.8 | Downregulated |
| **SOX8** | 16 | Promoter | Hypomethylation | 7 | Upregulated |
| **MEGF10** | 5 | Promoter | Hypermethylation | 7 | Downregulated |
| **RGS20** | 8 | Genebody | Hypermethylation | 6 | Upregulated |
| **RIMS4** | 20 | Promoter | Hypomethylation | 5.5 | Downregulated |
| **FERMT3** | 11 | Promoter | Hypomethylation | 5.3 | Upregulated |
| **IRX1** | 5 | Genebody | Hypermethylation | 5.2 | Downregulated |
| **KLF10** | 8 | Promoter | Hypermethylation | 4.9 | Downregulated |
| **CMSS1** | 3 | Genebody | Hypomethylation | 4.7 | Upregulated |
| **ZNF517** | 8 | Genebody | Hypermethylation | 4.6 | Downregulated |
| **SHC4** | 15 | Promoter | Hypermethylation | 4.6 | Downregulated |
| **RCOR2** | 11 | Promoter | Hypomethylation | 4.4 | Upregulated |
| **NECAB2** | 16 | Promoter | Hypomethylation | 4 | Upregulated |
| **FZD6** | 8 | Promoter | Hypermethylation | 4 | Downregulated |
| **DMRT2** | 9 | Genebody | Hypomethylation | 4 | Downregulated |
| **ZFP57** | 6 | Promoter | Hypomethylation | 3.8 | Upregulated |
| **PITX2** | 4 | Genebody | Hypermethylation | 3.7 | Downregulated |
| **ENPP2** | 8 | Promoter | Hypermethylation | 3.6 | Downregulated |
| **FAM195A** | 16 | Genebody | Hypermethylation | 3.5 | Upregulated |
| **ELFN1-AS** | 7 | Promoter | Hypomethylation | 3.4 | Upregulated |
| **TMC6** | 17 | Genebody | Hypermethylation | 3.2 | Upregulated |
| **RUSC2** | 9 | Genebody | Hypermethylation | 2.9 | Upregulated |
| **LINC01234** | 12 | Genebody | Hypomethylation | 2.9 | Downregulated |
| **ZC3H3** | 8 | Genebody | Hypermethylation | 2.8 | Upregulated |
| **FLYWCH** | 16 | Genebody | Hypermethylation | 2.8 | Upregulated |
| **COL9A3** | 20 | Genebody | Hypermethylation | 2.8 | Upregulated |
| **EIF2B5** | 3 | Genebody | Hypomethylation | 2.8 | Downregulated |
| **ARHGAP21** | 10 | Promoter | Hypermethylation | 2.7 | Downregulated |
| **TNFRSF1B** | 1 | Promoter | Hypomethylation | 2.6 | Upregulated |
| **SLC9A3** | 5 | Genebody | Hypomethylation | 2.6 | Downregulated |
| **EFS** | 14 | Promoter | Hypermethylation | 2.5 | Downregulated |
| **SGK1** | 6 | Promoter | Hypomethylation | 2.3 | Upregulated |
| **MROH6** | 8 | Genebody | Hypermethylation | 2.3 | Upregulated |
| **NAT14** | 19 | Genebody | Hypermethylation | 2.3 | Upregulated |
| **MAP4** | 3 | Genebody | Hypomethylation | 2.3 | Downregulated |
| **SLC22A20** | 11 | Genebody | Hypomethylation | 2.2 | Downregulated |
| **RAB40C** | 16 | Genebody | Hypermethylation | 2.2 | Upregulated |
| **IFT140** | 16 | Genebody | Hypermethylation | 2.2 | Upregulated |
| **CACNA1H** | 16 | Genebody | Hypermethylation | 2.2 | Upregulated |
| **IGSEQ1** | 3 | Genebody | Hypomethylation | 2.2 | Downregulated |
| **ZNF296** | 19 | Promoter | Hypomethylation | 2.1 | Upregulated |
| **KIFC2** | 8 | Genebody | Hypermethylation | 2.1 | Upregulated |
| **PARP10** | 8 | Genebody | Hypermethylation | 2.1 | Upregulated |
| **CHD7** | 8 | Genebody | Hypermethylation | 2.1 | Upregulated |
| **EFEMP2** | 11 | Promoter | Hypermethylation | 2.1 | Downregulated |
| **RBM15B** | 3 | Genebody | Hypomethylation | 2.1 | Downregulated |
| **AC024560.2** | 3 | Genebody | Hypomethylation | 2.1 | Downregulated |
| **WNK2** | 9 | Promoter | Hypomethylation | 2 | Upregulated |
| **BAIAP2** | 17 | Genebody | Hypermethylation | 2 | Upregulated |
| **PCDHA1-13** | 5 | Genebody | Hypomethylation | 2 | Downregulated |
| **ZBTB47** | 3 | Genebody | Hypomethylation | 2 | Downregulated |

| **Gene** | **Chromosome** | **Location DMR** | **Type of methylation** | **FC DMR** | **Expression** |
| --- | --- | --- | --- | --- | --- |
| **RCN1** | 11 | Genebody | Hypermethylation | 17 | Upregulated |
| **HOXA11-AS** | 2 | Genebody | Hypermethylation | 5.2 | Upregulated |
| **ALX3** | 1 | Promoter | Hypomethylation | 4.1 | Upregulated |
| **KLF11** | 3 | Promoter | Hypomethylation | 3.5 | Upregulated |
| **HES5** | 1 | Promoter | Hypomethylation | 3.3 | Upregulated |
| **ZNF578** | 19 | Genebody | Hypermethylation | 3.3 | Upregulated |
| **NFIC** | 19 | Genebody | Hypomethylation | 3 | Downregulated |
| **C2orf70** | 2 | Promoter | Hypomethylation | 2.9 | Upregulated |
| **SEMA3B** | 3 | Genebody | Hypermethylation | 2.9 | Upregulated |
| **KIF25** | 6 | Genebody | Hypomethylation | 2.7 | Downregulated |
| **ITGA5** | 12 | Promoter | Hypermethylation | 2.6 | Downregulated |
| **SLC9A3R2** | 16 | Genebody | Hypomethylation | 2.5 | Downregulated |
| **PCAT7** | 9 | Promoter | Hypomethylation | 2.4 | Upregulated |
| **TMEM151B** | 6 | Genebody | Hypermethylation | 2.1 | Upregulated |
| **AGPAT4** | 6 | Genebody | Hypomethylation | 2 | Downregulated |

**Supplementary Table 3.** SF3B1-specific DMRs that cause a significant gene expression change

**Supplementary Figure 3.** Boxplots showing the read count level, which indicates the level of methylation in all primary UM and all UM metastases.

**
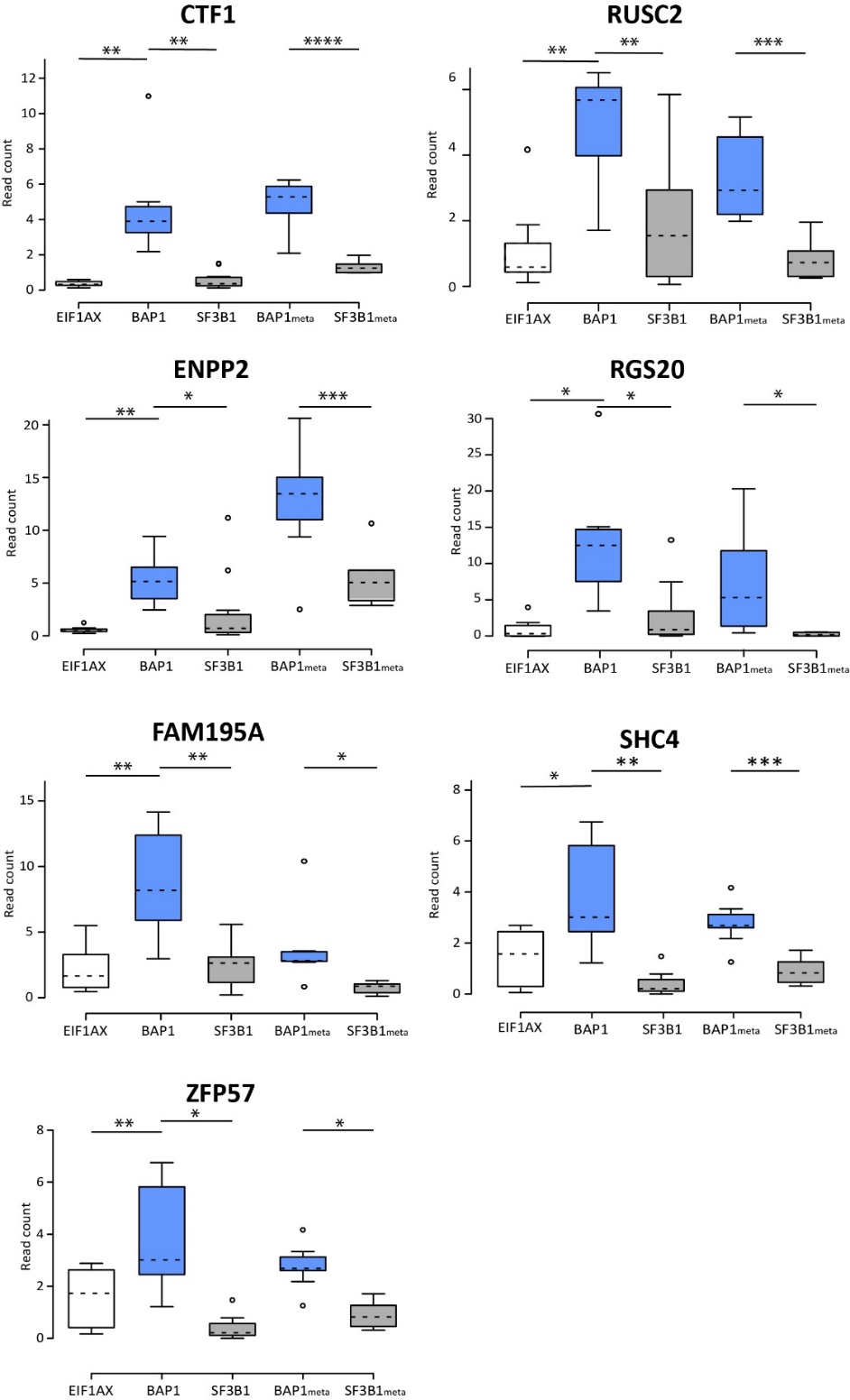
**
